# Seasonal influence on TORCH infection and analysis of multi‐positive samples with indirect immunofluorescence assay

**DOI:** 10.1002/jcla.22828

**Published:** 2019-01-21

**Authors:** Lu Chen, Jingrui Liu, Lei Shi, Yang Song, Yujie Song, Yang Gao, Ying Dong, Lin Li, Min Shen, Yanhong Zhai, Zheng Cao

**Affiliations:** ^1^ Department of Laboratory Medicine, Beijing Obstetrics and Gynecology Hospital Capital Medical University Beijing China; ^2^ Department of Laboratory Medicine, Cancer Hospital Chinese Academy of Medical Sciences Beijing China; ^3^ Central Laboratory, Beijing Obstetrics and Gynecology Hospital Capital Medical University Beijing China; ^4^ Reference Laboratory Medical System Biotechnology Co., Ltd. Ningbo China

**Keywords:** IFA, multipositive, prevalence, season, TORCH

## Abstract

**Background:**

TORCH including the pathogens of Toxoplasma gondii (TOX), rubella virus (RV), cytomegalovirus (CMV), and herpes simplex virus (HSV) causes intrauterine infections and poses a worldwide threat to women especially in pregnancy. In this study, we described the seasonal difference in TORCH infection and analyzed the anti‐TORCH IgM multipositive serum samples by the indirect immunofluorescence assays (IFA).

**Methods:**

To observe the seasonal influence of the anti‐TORCH IgG and IgM antibodies, a retrospective study was conducted with 10 669 women (20–40 y old) before pregnancy from August 2016 to July 2017. Totally 199 ELISA anti‐TORCH IgM multipositive serum samples were further tested by IFAs for false‐positive analysis.

**Results:**

The prevalence of positive HSV1‐IgM, RV‐IgM, HSV2‐IgM, CMV‐IgM, and TOX‐IgM in the present population was 6.30%, 2.55%, 1.94%, 1.24%, and 0.67%, respectively. Additionally, the prevalence of positive RV‐IgM, CMV‐IgM, and HSV1‐IgM was statistically different among four seasons, with the highest positive rates of RV‐IgM (4.12%) in autumn, CMV‐IgM (1.75%) in summer, and HSV1‐IgM (7.53%) in winter. The confirmatory IFAs showed that the positive rates of RUV‐IgM, CMV‐IgM, and HSV2‐IgM were significantly different from those in ELISA screening experiments. Interestingly, only 32.7% (65/199) of the TORCH IgM multipositive results were consistent with those by the IFA, indicating that cross‐reaction caused false positives were common in ELISA IgM antibody screening.

**Conclusion:**

The TORCH infection displayed different prevalence among four seasons in our 12‐month retrospective study. The IgM multipositives by ELISA screening may need further confirmation analysis due to its relatively high cross‐reaction rate.

## 1 INTRODUCTION

TORCH is an acronym that stands for a group of pathogens including Toxoplasma gondii (TOX), rubella virus (RV), cytomegalovirus (CMV), and herpes simplex virus (HSV) causing perinatal infections with similar symptoms.^1^ TOX is a common protozoan parasite in the phylum Apicomplexa, which is usually asymptomatic or in the form of a mild, self‐limiting illness characterized by fever, malaise, and lymphadenopathy. Nevertheless, in immunodeficiency or when congenitally acquired, it can result in severe disease even death if not treated properly.^2^ RV can lead to a devastating congenital rubella syndrome (CRS) of which manifestations are variable with deafness being most common.^3^ CMV infections may cause widespread dissemination throughout the body, with diverse cell types such as epithelial, endothelial, fibroblast, and smooth muscle cells supporting productive viral infection.^4^ The three major forms of neonatal HSV infection are disseminated disease, central nervous system (CNS) disease, and skin, eye, and mouth diseases.^5^

The serum TORCH antibody screening is crucial for early infection diagnosis,^6^ of which the IgM antibody detection is considered as the primary approach for acute and present infection. Due to the multiplex nature of the TORCH immunoassays implemented in clinical laboratories, it is not uncommon to have IgM multipositive results indicating potential co‐infections of multiple TORCH pathogens. Considering the cross‐reactions between antibodies and antigens are inevitable in immunoassays sometimes, careful scrutiny and clinical evaluations are required for patients with TORCH IgM multipositive results, to rule out potential false positives.

In this study, a twelve‐month retrospective evaluation with 10 669 women at childbearing age for the TORCH screening assays was conducted, to find out any seasonal influence on the infection rates. In addition, two serum samples that were tested TORCH IgM multipositive by enzyme‐linked immunosorbent assay (ELISA) were confirmed and analyzed by the “gold‐standard” indirect immunofluorescence assay (IFA).

## 2 MATERIALS AND METHODS

### 2.1 Subjects

The TORCH screening results of 10 669 women before pregnancy in Beijing Obstetrics and Gynecology Hospital from August 2016 to July 2017 were used in seasonal analysis. In the period of January 2016 to July 2017 during which 19 652 women were screened for before‐pregnancy TORCH infection screening, a total of 199 serum samples that were tested IgM multipositive from the TORCH assays were collected and saved at −80°C.

### 2.2 Reagents and methods

The TORCH ELISA screening assays were performed on the TECAN Freedom EVOlyzer® (Switzerland) platform with the TORCH IgM/IgG detection kits obtained from Trinity, TX, USA (Captia^TM^ TOX IgM/IgG; Captia^TM^ Rubella IgM/IgG; Captia^TM ^CMV IgM/IgG; Captia^TM ^HSV 1 IgM/IgG; Captia^TM ^HSV 2 IgM/IgG).

The IFA experiments were performed with a fluorescence microscope (OLYMPUS BX41, Japan). The TORCH IFA reagents used were listed as follows: IgM Indirect Fluorescence Kits (TOX Cat. I‐TOX01M, HSV1 Cat. I‐HSV01M and HSV2 Cat. I‐HSV01M) from SCIMEDX CORPORATION (Dover, NJ, USA); IgM Indirect Fluorescent Kits (RV Cat. 902701 and CMV Cat. 902123) from Hemagen (Columbia, MD, USA).

The ELISA experiments were performed according to the manufacturer’s instructions. Briefly, after the sample incubation step, the anti‐TORCH IgG/IgM antibodies in serum samples were bound to the solid phase with the TORCH‐specific antigens already attached to the surface of each reaction well. After excess antibody was removed by the washing buffer, the goat anti‐human IgG/IgM conjugated with horseradish peroxidase was added to the antibody‐antigen complexes, followed by the addition of the chromogen (tetramethylbenzidine). The relative color intensity indicating the concentration of the antibody in a patient serum was measured on an ELISA plate reader at the wavelength of 450 nm. The ELISA results were interpreted according to the manufacturer’s instructions, with signal/cutoff ratio greater than 0.9 as positive readings.

**Figure 1:**
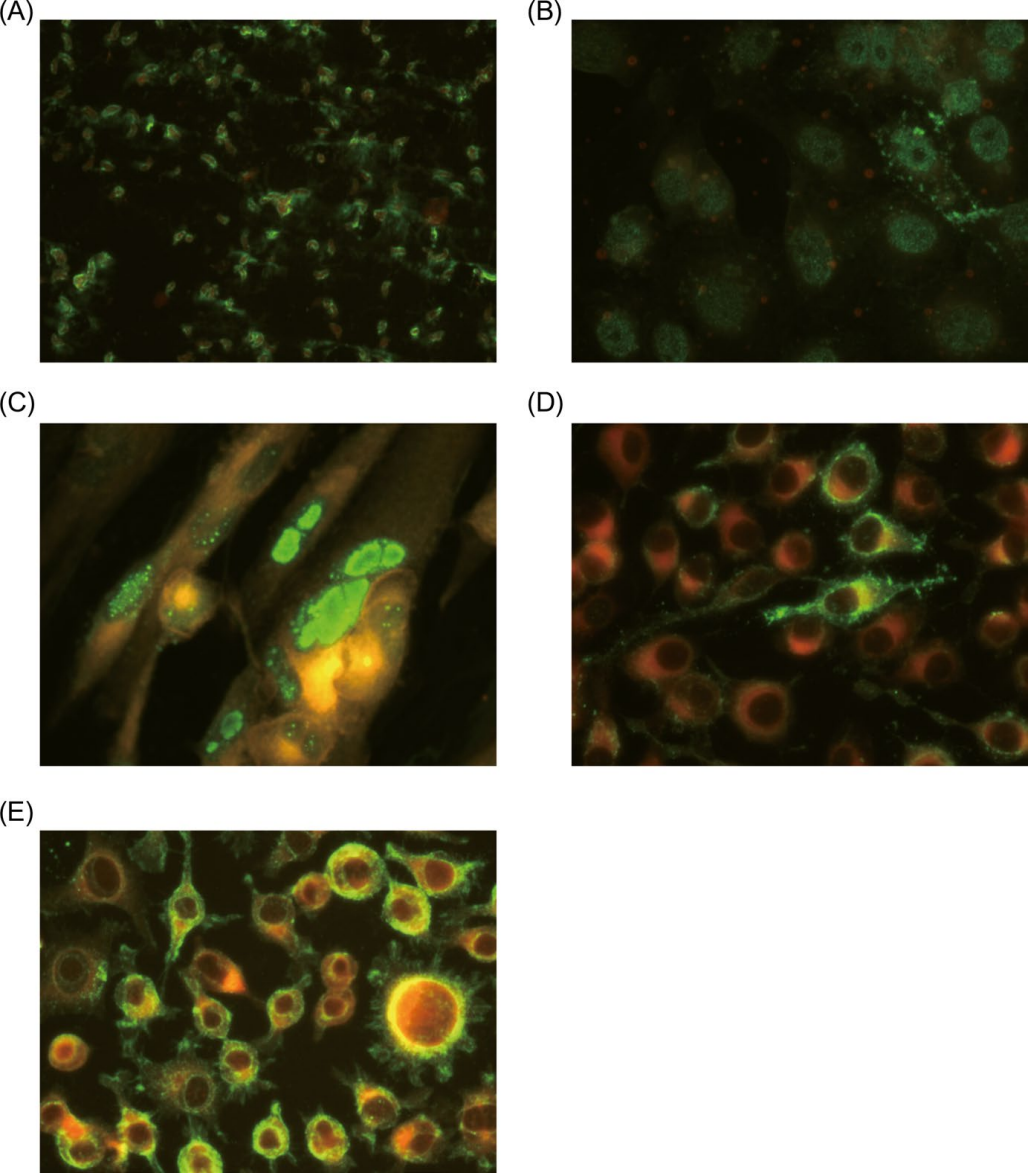
Examples of the positive TORCH IFA results from our patients at the magnification of 500‐fold. A, B, C, D, and E are anti‐TOX‐IgM, anti‐RV‐IgM, anti‐CMV‐IgM, anti‐HSV1‐IgM, and anti‐HSV2‐IgM‐positive IFA images, respectively

The experimental protocol for the IFA assays was described briefly as follows. Cover each reaction well with PBS‐diluted samples or controls (10–20 μl per well). After incubation in a humidified chamber at 20‐25°C, rinse the slides thoroughly in a staining dish of PBS and change the buffer and wash for an additional 8 minutes. Cover each well with fluorescein‐conjugated goat anti‐human IgM. After incubation and washing steps as described above, place a small drop of buffered glycerol in each well and cover with a coverslip, then the slides were read immediately at a magnification of 500‐fold, and a positive reaction for TORCH antibodies was indicated by the presence of diffuse nuclear inclusion bodies that emit green fluorescence (Figure 1).

### 2.3 Statistical analysis

SPSS 21.0 was used for data analysis (IBM Corporation. Chicago, United States). TORCH IgM infection differences were calculated by chi‐square test; two‐sided *P *< .05 was considered significant.

## 3 RESULTS

### 3.1 TORCH screening results in childbearing age women before pregnancy

With the ELISA screening assays described above, the positive rates of serum IgM antibodies to TORCH are listed in the order of decreasing prevalence: HSV1 (6.30%), RV (2.55%), HSV2 (1.94%), CMV (1.24%), and TOX (0.67%). As the IgM positivity is a strong indicator for primary infection, the TORCH IgG prevalence reflecting past exposure or vaccination was much higher than IgM, with CMV (92.05%), RV (89.74%), and HSV1 (81.56%) as the top prevalence IgG antibodies in the study population. See Table 1 for more details.

**Table 1:** TORCH ELISA results of 10 669 women before pregnancy

Pathogens	IgM		IgG	
	No. of positive	Positive rate (%)	No. of positive	Positive rate (%)
TOX	72	0.67	270	2.53
RV	272	2.55	9574	89.74
CMV	132	1.24	9821	92.05
HSV‐1	672	6.3	8702	81.56
HSV‐2	207	1.94	507	4.75

### 3.2 Seasonal influence on TORCH IgM positive rates

To find out whether the TORCH primary infection in our patients was different among four seasons, statistical analysis (chi‐square test, *P *< .05) was applied to the following study (Table 2) in which the TORCH IgM screening results were collected from a continuous 12‐month period (from August 2016 to July 2017). The positive rates of RV‐IgM, CMV‐IgM, and HSV1‐IgM were statistically different among four seasons. More specifically, the anti‐RV‐IgM and anti‐HSV1‐IgM showed a higher prevalence in autumn and winter, whereas the anti‐CMV‐IgM had the highest infection rate in summer (Table 2).

**Table 2:** Seasonal TORCH‐IgM positive rates

Antibodies	Total (n = 10 6699)	Winter (n = 2576)	Spring (n = 3312)	Summer (n = 2113)	Autumn (n = 2668)	*χ* ^2^	*P*
TOX‐IgM	72 (0.67)	14	26	11	21	2.515	0.473
RV‐IgM	272 (2.55)	93	29	40	110	79.27	<0.001
CMV‐IgM	132 (1.24)	21	44	37	30	8.824	0.032
HSV1‐IgM	672 (6.30)	194	190	95	193	23.99	<0.001
HSV2‐IgM	207 (1.94)	50	54	56	47	7.717	0.052

Notes

Winter: December, January, and February; spring: March, April, and May; summer: June, July, and August; autumn: September, October, and November.

### 3.3 False‐positive analysis in TORCH IgM multipositive samples.

According to the preliminary TORCH screening results, out of the 19 652 patients, totally 199 were tested IgM Multipositive. To confirm those multipositive infections, all the 199 serum samples were further analyzed by TORCH‐specific IgM IFA experiments, which are considered as “gold‐standard” assay in TORCH infection laboratory diagnosis. Of the 199 specimens retested by IFA, only 32.7% (65/199) had consistent results with ELISA experiments. In other words, as much as 67.3% of the specimens had one or more false‐positive IgM reactions by ELISA. No false negatives were identified in the TORCH IFAs when compared with the corresponding ELISA results (Table S1). To make data more comparable between the ELISA and the IFA experiments, the positive rates of TOX‐IgM, RV‐IgM, CMV‐IgM, HSV1‐IgM, or HSV2‐IgM were calculated as the number of each species‐specific positive IgM results divided by the sum of all positive IgM results from the 199 multipositive IgM patients (n = 598). As summarized in Table 3, the confirmatory IFA results of RV‐IgM, CMV‐IgM, and HSV2‐IgM were statistically different from those of primary screening (ELISA) (chi‐square test, *P *< .05), suggesting the false‐positive of these antibodies with ELISA should not be neglected. Based on the IFA experiments (Table 1), the most common co‐infection pattern was HSV1+HSV2, followed by TOX + HSV1 + HSV2 and CMV + HSV1 + HSV2. The cross‐reaction between anti‐HSV1 and anti‐HSV2‐IgM antibodies in ELISA was common when compared with IFA results (Table S1).

**Table 3:** ELISA and IFA results of 199 multipositive samples

Antibodies	ELISA positive	IFA positive	Total ELISA positive	ELISA positive %	IFA positive %	*χ* ^2^	*P*
TOX‐IgM	64	57	598	10.70	9.53	0.451	0.565
RV‐IgM	48	14	598	8.03	2.34	19.665	<0.001
CMV‐IgM	114	44	598	19.06	7.36	35.733	<0.001
HSV1‐IgM	189	173	598	31.61	28.93	1.014	0.345
HSV2‐IgM	183	144	598	30.60	24.08	6.402	0.014

## 4 DISCUSSION

As seen in Table 1, the anti‐TORCH IgG positive rates were much higher than those of IgM, especially for CMV‐IgG (92.05%), RV‐IgG (89.74%), and HSV‐1 IgG (81.56%) representing the past infection or vaccination history. As people can obtain life‐long immunity due to RV and TOX‐IgG antibodies, our results suggest that most women in our population had the resistance to RV. Both anti‐TOX‐IgM and IgG had the lowest prevalence, indicating minimum infection or exposure history. Domestic cats are only known hosts in which TOX can reproduce and complete its life cycles. Human gets infected through the ingestion of undercooked or raw meat, contaminated food or soil, transfusion of contaminated blood products, or organ transplantation. Vilibic‐Cavlek’s reports showed that the seroprevalence of TOX‐IgG in Croatian pregnant women was much higher (33.3%) than our data (2.53%).^7^ The possible reason could be that undercooked or even raw meat is more popular and acceptable in Europe than in China. In our study, the prevalence of TOX‐IgM was 0.67%, significantly lower than that (3.9%) reported by Feng in 2009,^8^ which may result from the improvement in citizens’ hygiene habits in recent years in Beijing or the theoretically higher sensitive of the chemiluminescent immunoassay method used in Feng’s study. Besides, Feng found that the TOX primary infection occurred more often in autumn and winter.^8^ Nevertheless, our study showed no significant seasonal difference in TOX‐IgM prevalence. To avoid congenital defects, it is advised that nonpregnant woman with an acute TOX infection should wait for at least 6 months before being pregnant.^9^

The CMV‐IgG had the highest prevalence in this study with 92.05%, which was consistent with the data reported previously, 60% in developed countries and almost 100% in developing countries.^10^ In other areas of China, CMV infection was also reported to have the highest prevalence in Baotou, Yan’an, and Zhengzhou.^11^, ^12^, ^13^ With regard to the TORCH seasonal difference, Feng’s article showed that CMV‐IgM infection was higher in summer and autumn, whereas it was found higher in spring and winter according to another study with women from Shandong province of China, suggesting the impact of geographic locations in CMV infection.^14^ Interestingly, we also observed a higher CMV‐IgM prevalence in spring and summer. By contrast, Formica’s group drew the conclusion that there was no significant difference of CMV prevalence among four seasons in 2012.^15^ Therefore, a nation‐wide study covering different regions of China is perhaps required in order to make definitive conclusions, if possible, about seasonal influence in TORCH infection in women before pregnancy. Formica’s group eventually draw the conclusion that there was no significant difference of CMV prevalence among seasons in 2012.^15^

The HSV1‐IgM prevalence was the highest in TORCH in this study (6.3%), consistent with Feng’s and Su’s findings about the women in Beijing^8^^,^ 16 and Gao’s in Shandong.^14^ Worldwide, the seroprevalence of herpes simplex is high: Over 33% of the world’s population has clinically recurrent HSV infections.^17^ HSV1 spreads through the incomplete skin and mucous membranes, as well as sexual contact which is considered as an increasing risk factor that contributes to its transmission. Data in this study showed the HSV2‐IgG positive rate (4.75%) was lower than previous reports (27.86% in Baotou and 14.64% in Beijing of 2015).^11^, ^16^ To rule out human errors and to further confirm our observation, the HSV2‐IgG positive rate for the last four months (from July in 2018 to October in 2018) was analyzed and similar HSV2‐IgG seroprevalence (5.49%) was observed. The HSV2‐IgG seroprevalence variation may be due to the differences in population (for instance, cities *vs* rural areas) and methodologies. The risk of vertical transmission of HSV from mothers with a primary HSV infection to fetuses is about 25–50% and decreases to less than 3% in women with a recurrent HSV infection due to placental protecting IgG antibody.^18^ If needed, cesarean section should be considered to avoid newborn infection by the birth canal.^5^ Similar to CMV infection, different seasonal prevalence observations were made in HSV1‐IgM. In spite of Su’s study in which the highest HSV‐IgM seroprevalence was found in summer, our data presented an opposite result that HSV1‐IgM showed lowest prevalence in summer. There are also reports about the HSV infection which showed no seasonal variation.^19^

As seen in Table 1, the RV‐IgM had the second highest prevalence (2.55%) in the TORCH ELISA screening experiments. This pathogen infects human through the respiratory tracts, which may explain for its seasonal distribution with the higher prevalence in autumn and winter during which Beijing usually has long‐lasting draught. Our finding is similar to Feng’s observation of RV‐IgM prevalence in 2009.^8^ Although the advent of vaccine has decreased the incidence of RV infection and CRS dramatically,^20^ women are advised not to become RV‐vaccinated during pregnancy.^21^ The limitation of this study is that the data were collected from a 12‐month period; cumulative observation for years might provide a better understanding about the seasonal influence on TORCH infections.^22^

The false‐positive ELISA results were confirmed by the “gold‐standard” TORCH‐specific IFA experiments. Three pathogen‐specific IgM antibodies (RV‐IgM, CMV‐IgM, and HSV2‐IgM) were observed to have statistically significant cross‐reactions in the ELISA screening assays, according to Table 3. More specifically, when compared with TORCH‐specific IFAs, CMV‐IgM, HSV1‐IgM, and HSV2‐IgM showed significantly decreased specificities, ranging from 29.09% to 54.84% (Table 4). In theory, IgM antibodies are sometimes produced as a result of a nonspecific activation of the immunological response according to previous study.^1^ Our results suggested that the TORCH‐IgM‐positive ELISA results must be interpreted carefully, especially for those patients who have multipositive IgM records. Further confirmatory experiment such as TORCH IFA may be warranted to identify potential false IgM‐seropositive cases.

**Table 4:** Sensitivity and specificity of TORCH ELISA with 199 multipositive samples

Antibodies	TP	FN	TN	FP	Total	Sen. (%)	Spe. (%)
TOX‐IgM	57	0	135	7	199	100.00	95.07
RV‐IgM	14	0	151	34	199	100.00	81.62
CMV‐IgM	44	0	85	70	199	100.00	54.84
HSV1‐IgM	173	0	10	16	199	100.00	38.46
HSV2‐IgM	144	0	16	39	199	100.00	29.09

Notes

The sensitivity and specificity of TORCH ELISA experiments were calculated and compared to TORCH‐specific IFAs. TP: true positive; FN: false negative; TN: true negative; FP: false positive; Sen: sensitivity; Spe: specificity.

In our study, the most common TORCH co‐infection patterns were HSV1 + HSV2, TOX + HSV1 + HSV2 and CMV + HSV1 + HSV2. The anti‐HSV1 and anti‐HSV2‐IgM antibodies tended to cross‐react with each other relatively easily due to their similar antigenic determinants.^23^ Marawan’s article pointed that TOX‐IgM‐positive tests were associated with seropositivity to HSV1‐IgG and HSV2‐IgM antibodies and that TOX‐seropositive patients were 1.94 and 1.35 times more likely than seronegative patients to have previous exposure to CMV and HSV‐1, respectively, which could be caused by that these infections are all linked to the poor socioeconomic conditions.^24^ In the study by Rasti et al,^25^ TOX‐IgG + CMV‐IgM and TOX‐IgG + HSV‐IgG + CMV‐IgM were the most frequent TORCH co‐infection patterns in the patients with abortion experience. Interestingly, the apparent TORCH co‐infection rates (201/19 000) by ELISA in Rasti’s report were pretty much comparable to that in our data, suggesting the similarity of the ELISA TORCH IgM screening power.

As an essential part of decreasing the congenital diseases, TORCH‐specific antibody screening results should be evaluated with extra care due to the existence of high rates of false‐positive IgM reactions, to decrease the unnecessary psychological and economic burdens of patients.
